# Bisphenol A Diglycidyl Ether Induces Adipogenic Differentiation of Multipotent Stromal Stem Cells through a Peroxisome Proliferator–Activated Receptor Gamma-Independent Mechanism

**DOI:** 10.1289/ehp.1205063

**Published:** 2012-05-25

**Authors:** Raquel Chamorro-García, Séverine Kirchner, Xia Li, Amanda Janesick, Stephanie C. Casey, Connie Chow, Bruce Blumberg

**Affiliations:** 1 Department of Developmental and Cell Biology, and; 2 Department of Pharmaceutical Sciences, University of California, Irvine, Irvine, California, USA

**Keywords:** adipogenesis, BADGE, BPA, endocrine disruption, MSCs, obesogen, PPARγ

## Abstract

Background: Bisphenol A (BPA) and bisphenol A diglycidyl ether (BADGE), used in manufacturing coatings and resins, leach from packaging materials into food. Numerous studies suggested that BPA and BADGE may have adverse effects on human health, including the possibility that exposure to such chemicals can be superimposed on traditional risk factors to initiate or exacerbate the development of obesity. BPA is a suspected obesogen, whereas BADGE, described as a peroxisome proliferator–activated receptor gamma (PPARγ) antagonist, could reduce weight gain.

Objectives: We sought to test the adipogenic effects of BADGE in a biologically relevant cell culture model.

Methods: We used multipotent mesenchymal stromal stem cells (MSCs) to study the adipogenic capacity of BADGE and BPA and evaluated their effects on adipogenesis, osteogenesis, gene expression, and nuclear receptor activation.

Discussion: BADGE induced adipogenesis in human and mouse MSCs, as well as in mouse 3T3-L1 preadipocytes. In contrast, BPA failed to promote adipogenesis in MSCs, but induced adipogenesis in 3T3-L1 cells. BADGE exposure elicited an adipogenic gene expression profile, and its ability to induce adipogenesis and the expression of adipogenic genes was not blocked by known PPARγ antagonists. Neither BADGE nor BPA activated or antagonized retinoid “X” receptor (RXR) or PPARγ in transient transfection assays.

Conclusions: BADGE can induce adipogenic differentiation in both MSCs and in preadipocytes at low nanomolar concentrations comparable to those that have been observed in limited human biomonitoring. BADGE probably acts through a mechanism that is downstream of, or parallel to, PPARγ.

Bisphenol A (BPA) is used in the synthesis of polycarbonate plastics, epoxy adhesives, and the lining of food containers. Bisphenol A diglycidyl ether (BADGE) is a synthesis product of BPA and epichlorhydrin used in the manufacture of epoxy resins, paints, and as a coating on food containers. BPA and BADGE are present in many commonly used products including beverage containers, baby bottles, and dental composites. Both migrate from containers into foods, and are routinely ingested (Cabado et al. 2008; Cao et al. 2009). Studies of BADGE metabolism suggest that it is not a significant source of BPA (Climie et al. 1981) although BPA leaches from some BADGE-containing dental sealants (Joskow et al. 2006; Olea et al. 1996).

BPA is an environmental endocrine-disrupting chemical (EDC) found in 95% of human urine samples (Calafat et al. 2008) as well as in serum, breast milk, and fat (reviewed by Rubin 2011; Taylor et al. 2011). Despite some controversy, the prevailing view in the scientific community is that BPA has important, deleterious effects in animals by acting on multiple target tissues (reviewed by Rubin 2011; Taylor et al. 2011), and BPA levels have been associated with adverse health outcomes in humans (Lang et al. 2008).

Estrogenic and antiandrogenic effects of BADGE have been reported (Olea et al. 1996; Satoh et al. 2004); however, the manufacturers of BADGE have disputed any endocrine-disrupting, oncogenic, or mutagenic effects (Poole et al. 2004). BADGE and its chlorohydroxy derivatives induced proliferation of human breast cancer cells but did not bind to the estrogen receptor (Nakazawa et al. 2002). BADGE exposure caused developmental toxicity during gestation and lactation in rats (Hyoung et al. 2007) and toxicity in cell culture (Ramilo et al. 2006). Overall, the presence of BADGE in food cannot be considered a health benefit and may be a health risk.

Obesity is caused by complex interactions among genetic, behavioral, and environmental factors, and EDC exposure is now thought to be a risk factor for obesity (reviewed by Janesick and Blumberg 2011; La Merrill and Birnbaum 2011; Tang-Peronard et al. 2011). Our “obesogen hypothesis” proposes a link between developmental EDC exposure and obesity. Obesogens are functionally defined as chemicals that promote obesity by increasing the number of fat cells (and fat storage into existing fat cells) by changing the amount of calories burned at rest, by altering energy balance to favor storage of calories, and by altering the mechanisms through which the body regulates appetite and satiety (reviewed by Janesick and Blumberg 2011).

Most evidence suggests that BPA acts as an obesogen, *in vitro* and *in vivo*. BPA induced adipocyte differentiation and adipogenic marker genes in 3T3-L1 preadipocytes (Masuno et al. 2005). Perinatal treatment of rats (Rubin et al. 2001; Somm et al. 2009) and mice (Miyawaki et al. 2007) with low doses of BPA led to increased fat mass (reviewed by Rubin 2011). Some studies suggested that different BPA dosing regimens might not increase body weight in rats (Nunez et al. 2001; Seidlova-Wuttke et al. 2005) or mice (Ryan et al. 2010), and thus further studies are needed to clarify exactly how BPA promotes adipogenesis and obesity.

The obesogenic properties of BADGE are yet to be thoroughly investigated. BADGE was identified as an antagonist of peroxisome proliferator–activated receptor gamma (PPARγ) [its IC_50_ (the concentration of BADGE at which 50% inhibition of the response is observed) is approximately 100 µM] that also inhibits differentiation of 3T3-L1 and 3T3-F442A preadipocytes (Wright et al. 2000). BADGE administered orally at high doses to mice on a high-fat diet decreased triglyceride content in white adipose tissue, skeletal muscle, and the liver due to increased leptin (LEP) effects and increased fatty acid combustion and energy dissipation, thereby ameliorating high-fat diet–induced obesity and insulin resistance (Yamauchi et al. 2001; Yun et al. 2008). In contrast, BADGE induced nuclear localization and activation of PPARγ leading to apoptosis in ECV304 cells (Bishop-Bailey et al. 2000), whereas BADGE induced apoptosis in different tumor cell lines in a PPARγ-independent manner (Fehlberg et al. 2002). Therefore, the effects of BADGE on PPARγ, adipogenesis, and other cellular processes may be cell-type specific.

Most mechanistic studies of adipocyte differentiation have used murine preadipocyte cell lines such as 3T3-L1 and 3T3-F442A as models (Rosen and MacDougald 2006). Multipotent mesenchymal stromal stem cells (MSCs) are a useful model for studying changes in the programming of adipogenesis because they are the cells that give rise to adipocyte progenitors *in vivo* (Avram et al. 2007). We used this model to show that the organotin tribultyltin chloride (TBT), a PPARγ activator, can reprogram the fate of MSCs, predisposing them to differentiate into adipocytes (Kirchner et al. 2010). While studying a panel of chemicals for their effects on adipogenesis in MSCs, we unexpectedly found that BADGE elicited a dose-dependent conversion of human and mouse MSCs into adipocytes whereas BPA did not. Moreover, antagonizing PPARγ did not block the adipogenic effects of BADGE, and neither BADGE nor BPA activated or antagonized PPARγ. Unlike obesogens that act through PPARγ, BADGE did not alter the balance between the osteogenic and adipogenic capacities of MSCs. We conclude that BADGE is likely to be an obesogen that acts through a pathway downstream of, or parallel to, PPARγ.

## Methods

*Bone marrow stem cell culture.* Unless otherwise noted, all chemicals were purchased from Sigma-Aldrich (St. Louis, MO). MSCs were maintained as subconfluent monolayers in basic medium [Dulbecco’s Modified Eagle Medium (DMEM; GIBCO-BRL, Gaithersburg, MD) containing 10% calf bovine serum (Premium Select; Atlanta Biologicals, Atlanta, GA), 100 IU/mL penicillin, 100 μg/mL streptomycin, and 1 mM sodium pyruvate] as described previously (Kirchner et al. 2010). Mouse bone marrow MSCs were purchased from Invitrogen (Carlsbad, CA). Human bone marrow mononuclear cells were purchased from Lonza (Walkersville, MD). Stromal cells showed an expression profile consistent with an MSC population and could differentiate into adipocytes, osteoblasts, and chondrocytes, and they will be referred to hereafter as MSCs.

*3T3-L1 cell culture.* 3T3-L1 cells were maintained in DMEM supplemented with 10% fetal bovine serum, 2 mM l-glutamine, 50 IU/mL penicillin, and 50 μg/mL streptomycin containing TBT or the PPARγ agonist rosiglitazone (ROSI) at the concentrations indicated in the figure legends. Culture conditions, adipogenic differentiation, and analysis were performed as described previously (Li et al. 2011). All experiments were repeated at least three times.

*Differentiation assays.* Cells were treated with fat, bone, or cartilage differentiation medium (for 14, 21, or 21 days, respectively) or with basic MSC expansion medium (yielding untreated cells for no-differentiation controls) as described previously (Kirchner et al. 2010). Adipose differentiation was also performed in the presence of the PPARγ antagonists T0070907 (100 nM) or GW9662 (500 nM), which were renewed every 8 hr (GW9662) or 12 hr (T0070907). Cells were stained with Oil-Red O to measure lipid accumulation and Alizarin Red to measure calcium deposition and were quantified using Image J imaging software, version 1.36b (W. Rasband) as described (Kirchner et al. 2010). Data represent mean ± SE from three independent experiments in duplicate.

*Transfection assays.* pCMX-GAL4 and fusion constructs to nuclear receptor ligand-binding domains (GAL4-hPPARγ, GAL4-hRXRα) were described previously (Grun et al. 2006). One microgram CMX-GAL4 effector plasmid was co-transfected with 5 µg CMX-β-galactosidase transfection controls and 5 µg tk-(MH100)_4_-luciferase reporter plasmids (per 96-well plate) into COS7 cells using Lipofectamine 2000 reagent (Invitrogen; Life Technologies, Carlsbad, CA) following the manufacturer’s recommended protocol. Briefly, COS7 cells were seeded at 15,000 cells per well in 96-well tissue culture plates in calf bovine serum and transfected the following day in Opti-MEM reduced serum medium at approximately 90% confluency. After overnight incubation, the medium was replaced with DMEM/10% resin charcoal-stripped fetal bovine serum (Tabb et al. 2004) plus ligands for an additional 24 hr before luciferase and β-galactosidase assays (Milnes et al. 2008). For activation assays, BADGE and BPA were tested from 10^–10^ M to 10^–4^ M, with 10^–4^ M producing noticeable cytotoxicity as judged by reduced β-galactosidase activity. The control compounds ROSI (PPARγ agonist) and LG-268 [retinoid “X” receptor (RXR) agonist] were tested from 10^–10^ M through 10^–5^ M. For antagonism assays, BADGE, BPA, GW9662 (PPARγ antagonist), or HX-531 (RXRα antagonist) were tested from 10^–10^ M through 10^–5^ M against 10^–7^ M ROSI (PPARγ) or 10^–7^ M LG-268 (RXRα). All transfections were performed in triplicate and reproduced in multiple experiments. Data are reported as fold induction over vehicle (0.1% DMSO) controls ± SE for activation assays or as fold reduction over 10^–7^ M ROSI or 10^–7^ M LG-268 ± SE for antagonism assays.

*Quantitative real-time reverse transcriptase polymerase chain reaction (QPCR).* Total RNA was extracted using the TRIzol reagent (GIBCO-BRL). cDNA was generated from 1 μg DNase-treated RNA using Transcriptor Reverse Transcriptase (Roche, Nutley, NJ) following the manufacturer’s protocol. QPCR analyses for target genes were performed with FastStart SYBR Green QPCR Master Mix (Roche) and 100 nM of primers [see Supplemental Material, Table S1 (http://dx.doi.org/10.1289/ehp.1205063)] chosen using PerlPrimer (version 1.1.14; copyright 2003–2006, O. Marshall) in a DNA Engine Opticon Thermal Cycler (MJ Research; Watertown, MA, Bio-Rad Laboratories, Hercules, CA). Relative quantification of the target gene transcript in comparison with β-actin (housekeeping gene) expression levels in the same sample followed the ΔΔC_t_ method (Livak and Schmittgen 2001).

*Flow cytometry.* Cultured human and mouse bone marrow MSCs that were induced and exposed to DMSO, ROSI, BADGE, or BPA were harvested and processed for staining and flow cytometry as described previously (Kirchner et al. 2010). FABP4 (fatty acid binding protein 4) was detected using phycoerythrin-conjugated streptavidin (eBioscience, San Diego, CA). Cells were analyzed using a FACSCalibur (Becton Dickinson, Franklin Lakes, NJ) running FlowJo software version 8.7.1 (Treestar, Ashland, OR) as described previously (Kirchner et al. 2010). Median fluorescence intensities (MFI) of FABP4 staining were normalized from three independent assays using DMSO treatment as the control.

*Statistical analyses.* Data are presented as mean ± SE. We used unpaired *t*-tests to determine the significance of the difference in relative mRNA abundance or staining among groups with different treatments. *p* < 0.05 was considered statistically significant. We used GraphPad Prism, version 5.0 (GraphPad Software, Inc., San Diego, CA), for statistical analysis.

## Results

In vitro *BADGE exposure enhances differentiation of MSCs into adipocytes.* We previously characterized a stromal cell population derived from white adipose tissue and showed that these cells could differentiate into fat, bone, or cartilage *in vitro* and had the properties expected for MSCs (Kirchner et al. 2010). In the present study, we tested the suitability of bone marrow–derived MSCs as models for adipogenesis and found that the gene expression profiles of the adherent cell population from human (hMSC) and C57BL6/J mouse (mMSC) bone marrow were consistent with expectations for an MSC-enriched population and differentiated into adipocytes, osteoblasts, and chondrocytes in culture [see Supplemental Material, Figure S1 (http://dx.doi.org/10.1289/ehp.1205063)].

**Figure 1:**
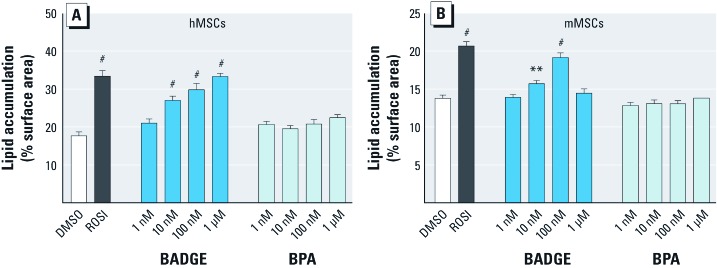
Dose response of MSCs adipogenic capacities to *in vitro* exposure to BADGE and BPA. Adipogenesis was induced in hMSCs (*A*) and mMSCs (*B*) by the addition of an adipogenic cocktail for 14 days in the absence (DMSO) or presence of ROSI 500 nM or increasing doses of BADGE and BPA. All data are expressed as mean ± SE lipid accumulation in six replicates. *p < 0.05, **p < 0.01, and ^#^*p* < 0.001 relative to vehicle (DMSO) controls.

When adipogenic differentiation was induced by treatment with the standard adipocyte induction cocktail (isobutylmethylxanthine, dexamethasone, insulin, indomethacin; MDII) and DMSO vehicle, lipid droplets covered approximately 18% of the dish surface in hMSCs and approximately 14% in mMSCs (Figure 1) [for representative micrographs and details of quantitation, see Supplemental Material, Figure S2 (http://dx.doi.org/10.1289/ehp.1205063)]. At 500 nM, ROSI caused a significant increase in lipid accumulation in both hMSCs and mMSCs (Figure 1). The known obesogen BPA was unable to induce adipogenesis in hMSCs and mMSCs at concentrations between 1 nM and 100 µM (Figure 1, and data not shown). Higher concentrations were cytotoxic. BADGE led to a significant increase in lipid accumulation at 10 nM in both hMSCs and mMSCs (Figure 1). We consistently found that hMSCs were more responsive to adipogenic stimulation than mMSCs.

**Figure 2:**
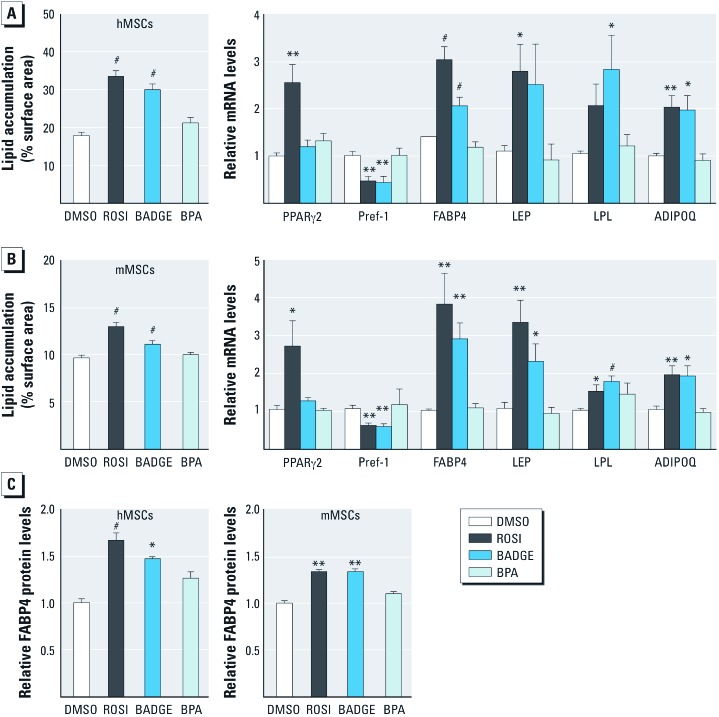
*In vitro *effect of BADGE exposure on the adipogenic capacities of hMSCs and mMSCs. Lipid accumulation in hMSCs (*A*) and mMSCs (*B*) (left). Adipogenesis was induced in MSCs by the addition of an adipogenic cocktail for 14 days with DMSO, 500 nM ROSI, 100 nM BADGE, or 100 nM BPA. Gene expression profile in hMSCs and mMSCs was assayed by QPCR (right) [early adipogenesis markers: PPARγ2, Pref-1, and FABP4; late adipogenesis markers: LEP, LPL, and ADIPOQ]. Expression was normalized to β-actin. (*C*) FABP4 protein levels were assayed by flow cytometry. Median fluorescence intensities (MFI) are represented relative to vehicle (DMSO) controls. ADIPOQ, adiponectin. All data are expressed as mean fold change ± SE in six replicates. *p < 0.05, **p < 0.01, and ^#^*p* < 0.001 relative to vehicle (DMSO) controls.

Subsequent experiments used 100 nM BADGE, a concentration that induced lipid accumulation to a level comparable with that of 500 nM ROSI in both hMSCs (Figure 2A) and mMSCs (Figure 2B). 100 nM BPA was used to test whether the effects of BADGE could be mediated by conversion to BPA. QPCR analysis of mRNA levels revealed an adipogenic profile in MSCs exposed to BADGE but not to BPA (Figure 2A,B right). The early adipogenesis marker FABP4 and the late markers LEP, lipoprotein lipase (LPL), and adiponectin (ADIPOQ) were significantly increased in mMSCs, whereas the adipogenesis inhibitor Pref-1 (adipocyte differentiation-associated protein-1/preadipocyte factor-1) was decreased, by treatment with 500 nM ROSI or 100 nM BADGE (Figure 2A,B). LEP induction by BADGE and LPL induction by ROSI showed strong trends in hMSCs but did not reach statistical significance (Figure 2A). BPA did not elicit any significant changes in gene expression (Figure 2A,B). To verify that the increased expression of mRNA was reflected at the protein level, we quantitated FABP4 protein using flow cytometric analysis of cells stained with an anti-FABP4 antibody (Kirchner et al. 2010). Both ROSI and BADGE elicited significant increases in FABP4 protein, whereas BPA had no significant effect (Figure 2C). Thus, BADGE, but not BPA, induced adipogenic differentiation in MSCs, suggesting that BADGE itself, rather than BPA derived from BADGE, was the responsible agent.

*Both BADGE and BPA induce adipogenesis in 3T3-L1 preadipocytes.* We were surprised that the known obesogen BPA (Rubin 2011) did not induce adipogenesis in MSCs, whereas BADGE did. Considering the obesogenicity of BPA in animals, we hypothesized that BPA might be able to stimulate adipogenesis in cells already committed to the adipocyte lineage, but not in MSCs. Therefore, we tested the effects of BADGE and BPA in 3T3-L1 preadipocytes. In contrast to the results in MSCs (Figures 1 and 2), and in accord with published studies (Masuno et al. 2005; Sargis et al. 2010), BPA induced adipogenesis in 3T3-L1 cells at 10 nM, with significant induction of triglyceride accumulation observed at 100 nM [see Supplemental Material, Figure S3A (http://dx.doi.org/10.1289/ehp.1205063)] and FABP4 expression at 10 nM (see Supplemental Material, Figure S3B). BADGE showed significant induction of adipogenesis at 10 nM (see Supplemental Material, Figure S3A,B). We infer that BADGE can induce both MSCs and preadipocytes to undergo adipogenesis, whereas BPA was only effective in preadipocytes.

**Figure 3:**
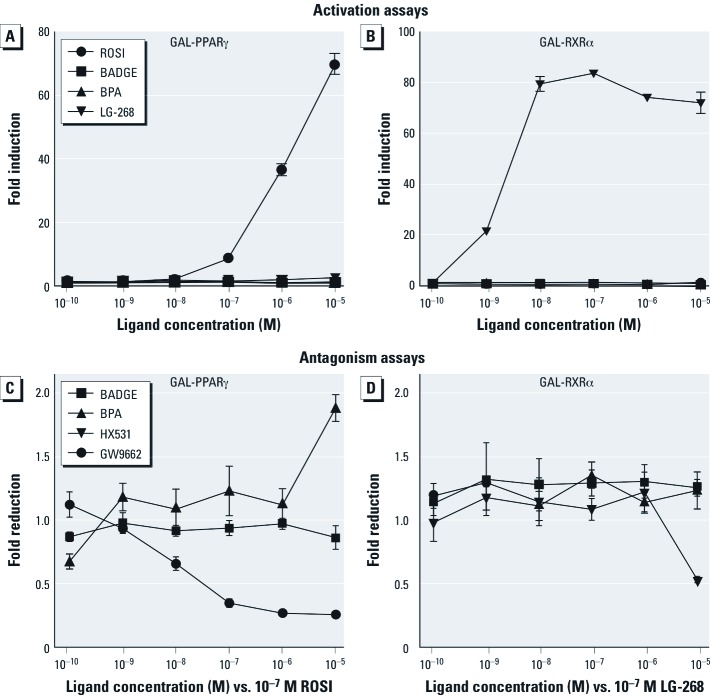
Neither BADGE nor BPA affects PPARγ or RXR activity in transient transfection assays. The ability of a graded dose series of BADGE or BPA to activate or antagonize GAL4-hPPARγ and GAL-hRXRα was tested in transiently transfected COS7 cells. (*A*,*B*) Activation assays: BADGE and BPA (10^–10^ M through 10^–4^ M) were tested with 10^–4^ M producing noticeable cytotoxicity as judged by β-galactosidase activity. The control compounds ROSI (PPARγ agonist) and LG-268 (RXR agonist) were also tested (10^–10^ through 10^–5^ M). Neither BADGE nor BPA activated PPARγ or RXR. (*C*,*D*) Antagonism assays: BADGE, BPA, GW9662 (PPARγ antagonist), and HX-531 (RXR antagonist) were tested (10^–10^ through 10^–5^ M) against 10^–7^ M ROSI (PPARγ; *C*) or 10^–7^ M LG-268 (RXRα; *D*). Although GW9662 effectively antagonized PPARγ activity, BADGE and BPA did not. HX-531 antagonized RXRα at 10^–5^ M, whereas BADGE and BPA were inactive. Data are presented as fold induction over vehicle (0.1% DMSO) controls for activation assays or as fold reduction over 10^–7^ M ROSI or 10^–7^ M LG-268 for antagonism assays.

*BADGE does not activate or antagonize PPAR*γ *or RXR.* Many EDCs mimic natural lipophilic hormones that act through members of the nuclear receptor superfamily (Diamanti-Kandarakis et al. 2009). Because PPARγ is believed to be the master regulator of adipogenesis (Tontonoz and Spiegelman 2008) and because BADGE has been shown to activate PPARγ in some cell types (Bishop-Bailey et al. 2000; Nakamuta et al. 2002), we hypothesized that BADGE might function by activating PPARγ or its heterodimeric partner, RXR. We tested the ability of ROSI, BADGE, BPA, and LG-268 (control RXR agonist) to activate PPARγ (GAL4-hPPARγ) or RXRα (GAL4-hRXRα) LBD constructs in transient transfection assays in COS7 cells. Whereas ROSI and LG-268 could fully activate PPARγ and RXRα respectively, neither BADGE nor BPA had any effect on PPARγ or RXRα activation at any dose tested (Figure 3A,B). We also tested the ability of BADGE to antagonize RXRα or PPARγ since it was previously shown that BADGE antagonized PPARγ with an IC_50_ of approximately 100µM (Wright et al. 2000). The PPARγ antagonists GW9662 (Figure 3C) and T0070907 (not shown) efficiently antagonized PPARγ activation by ROSI, whereas BPA and BADGE had no effect at ≤ 10 µM (Figure 3C). Higher doses of BADGE were toxic to COS7 cells in our experiments. The RXR antagonist HX-531 (Ebisawa et al. 1999) antagonized RXRα, whereas neither BADGE nor BPA had any antagonistic activity (Figure 3D).

*BADGE does not alter the balance of adipogenic versus osteogenic commitment in MSCs.* The reciprocal relationship between adipocyte and osteocyte lineage allocation in MSCs is well documented and involves a PPARγ-mediated shift in the flow of mesenchymal precursors from osteogenic to adipogenic lineages (Takada et al. 2007). When bone differentiation was induced by the osteogenic cocktail in MSCs, approximately 80% of the extracellular matrix surface was calcified in hMSCs [see Supplemental Material, Figure S4A (http://dx.doi.org/10.1289/ehp.1205063)], whereas approximately 22% was calcified in mMSCs (see Supplemental Material, Figure S4B). The addition of 500 nM ROSI during osteogenic differentiation resulted in a pronounced decrease in matrix calcification, whereas adding 100 nM BADGE had no effect (see Supplemental Material, Figure S4A,B). BPA had no effect in hMSCs (see Supplemental Material, Figure S4A), but caused a significant decrease in calcification in mMSC (see Supplemental Material, Figure S4B). Expression of mRNA encoding the bone markers alkaline phosphatase (ALP) and osteocalcin (OST) was significantly reduced by the addition of ROSI in both hMSCs (see Supplemental Material, Figure S4C) and mMSCs (see Supplemental Material, Figure S4D). Expression of the runt related transcription factor 2 (Runx2) was not modified and the osteogenic marker osteopontin (OPN) was decreased only in hMSCs (compare Supplemental Material, Figure S4C,D). BADGE did not affect mRNA levels for any of these markers, whereas BPA decreased ALP expression in mMSCs (see Supplemental Material, Figure S4C,D). Therefore, BADGE exposure does not promote or facilitate adipogenic differentiation to the detriment of osteogenic differentiation in MSCs, and we therefore infer that BADGE probably acts through a different mechanism than does ROSI or other PPARγ activators such as TBT.

**Figure 4:**
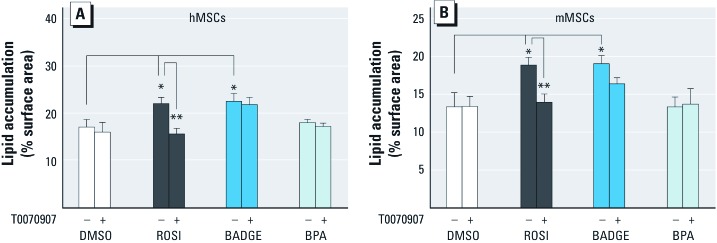
Effect of the PPARγ antagonist T0070907 in BADGE- or BPA-induced adipogenic abilities of MSCs. Adipogenesis was induced in hMSCs (*A*) or mMSCs (*B*) by the addition of an adipogenic cocktail for 14 days with DMSO, 500 nM ROSI, 100 nM BADGE, or 100 nM BPA in the presence of DMSO or 100 nM T0070907 (PPARγ antagonist). All data are expressed as mean ± SE lipid accumulation in six replicates. *p < 0.05, and **p < 0.01 relative to vehicle (DMSO) controls.

*Antagonizing PPAR*γ *does not block the adipogenic effects of BADGE in MSCs.* We next tested whether antagonizing PPARγ could block BADGE-induced adipogenesis. In the presence of 500 nM ROSI, adipogenic differentiation of both hMSCs and mMSCs was inhibited by the addition of the potent PPARγ antagonists T0070907 (Figure 4A) or GW9662 (not shown). In contrast, T0070907 had no effect on BADGE-mediated induction of adipogenesis in these cells, and BPA did not induce adipogenesis. We conclude that BADGE is not a significant activator of PPARγ and instead facilitates adipose conversion of the MSCs through a pathway that is unlikely to be PPARγ mediated.

## Discussion

A single risk factor is rarely responsible for the development of most chronic diseases. The factors driving obesity, diabetes, cardiovascular diseases, hypertension, and dyslipidemia are usually ascribed to genetics (Herbert 2008) and behaviors such as smoking (Power and Jefferis 2002) and excessive consumption of alcohol (Mantena et al. 2008) and food (Hill and Peters 1998), together with increased stress (Garruti et al. 2008), sedentary lifestyle (Rippe and Hess 1998), or infectious agents (Dhurandhar 2004). The environmental obesogen hypothesis proposes that exposure to EDCs may be superimposed on these conditions and exacerbate the development of obesity (reviewed by Janesick and Blumberg 2011). We previously showed that *in vitro* or *in utero* exposure to TBT can compromise the PPARγ-mediated balance between adipogenic and osteogenic lineages of MSCs, thereby altering the stem cell compartment to bias the MSC population toward the adipocyte lineage (Kirchner et al. 2010). In the present study, we used a similar cell model to investigate the obesogenic properties of BPA and BADGE. Surprisingly, we found that while the known obesogen BPA (Rubin and Soto 2009; Somm et al. 2009) could induce adipogenesis in 3T3-L1 preadipocytes, BPA was unable to induce adipogenesis in hMSCs or mMSCs. However, BADGE, which had previously been described as a PPARγ antagonist and inhibitor of adipogenesis (Wright et al. 2000), induced adipogenesis in both MSCs and preadipocytes though a mechanism that is not inhibited by highly potent and selective PPARγ antagonists. Considering that BPA was unable to induce adipogenesis in our assays, it is unlikely that the ability of BADGE to promote adipogenesis was due to BPA produced from BADGE metabolism or degradation.

Unfortunately, there is a paucity of data regarding the human urinary or plasma levels of BADGE, and the appropriateness of the concentrations used in animal studies compared to actual human exposure can only be estimated. No published studies measuring BADGE levels in humans are available. The Environmental Working Group (2005) informally surveyed 70 industrial chemicals in 22 volunteers from across the U.S. and reported a mean serum BADGE concentration of 17.3 ng/mL (approximately 51 nM) with a maximum of 174 ng/mL (approximately 512 nM). Health Canada estimated the maximum daily intake of BADGE in infants consuming soy formula at 22 µg/kg body weight (producing a mean body concentration of approximately 65 nM) (Cao et al. 2009). In contrast, computer models used by the European Commission suggest a daily intake of BADGE of 0.16 µg/kg (Holmes et al. 2005). In aqueous and acidic foodstuffs, BADGE readily generates mono- and dihydrolyzed products (Hammarling et al. 2000). BADGE and its derivatives were found at concentrations > 1 mg/kg in many canned foods; the highest concentration of BADGE observed was 12.5 mg/kg food (Biedermann and Grob 1998; Hammarling et al. 2000; Uematsu et al. 2001). The European Commission established an upper limit of 1 mg/kg (approximately 3 µM) in food as a temporary restriction for specific migration of the sum of BADGE and its hydrolysis products (European Commission 2002).

The published BADGE-induced decreases in rodent body weights were observed at concentrations (several hundreds of milligrams per kilogram administered daily) (Hyoung et al. 2007; Yun et al. 2008) that are unrealistically high compared to estimated human intakes. We found that BADGE promoted adipogenesis in hMSCs, mMSCs, and 3T3-L1 preadipocytes at nanomolar levels (Figs. 1–3). These levels are comparable to the average of approximately 51 nM observed in the limited human biomonitoring (Environmental Working Group 2005). Our data raise important questions about whether BADGE could potentially cause weight gain in human at biologically relevant doses.

The molecular mechanisms underlying our observations are unclear. BADGE was described as a PPARγ antagonist that blocked ligand-mediated adipocyte differentiation in 3T3-L1 and 3T3-F442 cell lines (Wright et al. 2000). Notably, BADGE was described as a low-affinity PPARγ ligand that required very high concentrations (IC_50_ of approximately 100 µM) to demonstrate PPARγ antagonism (Seimandi et al. 2005; Wright et al. 2000), concentrations that are unlikely to be achieved *in vivo*. We showed that BADGE could not activate or antagonize PPARγ at concentrations ranging from 1 nM to 10 µM, whereas higher concentrations were toxic.

In contrast to the reported effects of BADGE in preadipocyte cell lines noted above, we found that BADGE augmented, rather than inhibited, adipogenesis in 3T3-L1 preadipocytes and hMSCs and mMSCs (which more faithfully represent adipocyte progenitors *in vivo*). Moreover, the BADGE-induced adipogenic differentiation of hMSCs and mMSCs was not reduced by the addition of the potent PPARγ antagonists T0070907 or GW9662. This supported our hypothesis that PPARγ activation was not required for the ability of BADGE to induce adipogenesis, although a detailed crystallographic analysis of potential interactions between BADGE and PPARγ would be required to completely rule out the possibility that BADGE acts through PPARγ.

PPARγ expression and activation are important regulators of lineage allocation between adipogenic and osteogenic pathways in MSCs (Takada et al. 2007). We recently showed that the EDC TBT counteracted the induction of osteogenesis in MSCs, instead promoting adipogenesis (Kirchner et al. 2010). In contrast, although BADGE induced adipogenesis in hMSCs and mMSCs, it did not affect osteogenic differentiation. Since the balance between these two lineages was unaffected, it is unlikely that the regulator of this process, PPARγ, mediated BADGE action. Moreover, BADGE has been shown to have PPARγ-independent action on apoptosis in tumor cells (Fehlberg et al. 2002), supporting the idea that BADGE can have PPARγ-independent effects.

There is an urgent need to understand the mechanisms underlying the predisposition to obesity and related disorders. In the present study, we have identified unexpectedly potent effects of a ubiquitously used chemical, BADGE, on adipogenesis in MSCs at nanomolar levels. While exposure data are currently limited, this is in the same range as reported human exposures. Therefore, it will be essential to determine the levels of BADGE and its routes of exposure, metabolism, and retention in humans. Future *in vivo* studies should also test the effects of BADGE exposure at biologically realistic concentrations. Lastly, considering the recent popularity of testing chemical toxicity by high throughput screening (e.g., ToxCast, Tox21) (Dix et al. 2007; Shukla et al. 2010) and the different results of BPA on MSCs and preadipocytes, we suggest that cell-based testing of chemicals for adipogenic properties in MSCs should be considered in the hunt for environmental obesogens.

## Supplemental Material

(2.8 MB) PDF
