# Supplementary material for: Bisphenol A Diglycidyl Ether Induces Adipogenic Differentiation of Multipotent Stromal Stem Cells through a Peroxisome Proliferator–Activated Receptor Gamma-Independent Mechanism
Source: Environ Health Perspect. 2012 May 25;120(7):984–9. doi: 10.1289/ehp.1205063 (PMC3404682; doi:10.1289/ehp.1205063)
Supplement: (2.8 MB) PDF [file ehp.1205063.s001.pdf]

Supplemental Material - Bisphenol A Diglycidyl Ether Induces Adipogenic Differentiation of Multipotent Stromal Stem Cells through a Peroxisome Proliferator Activated Receptor Gamma-independent Mechanism

Raquel Chamorro-García[1,#], Séverine Kirchner[1,#], Xia Li[1], Amanda Janesick[1], Stephanie C. Casey[1], Connie Chow[1], and Bruce Blumberg[1, 2]

[1]Department of Developmental and Cell Biology, 2011 Biological Sciences 3, [2]Department of Pharmaceutical Sciences, University of California, Irvine, CA 92697-2300, US.

[#]These authors contributed equally to this work

Supplemental Material - Table of Contents

**Item**	**Page**
Supplemental Table S1	3
Supplemental Figure S1	4-5
Supplemental Figure S2	6
Supplemental Figure S3	7
Supplemental Figure S4	8-9
References Cited	10

Supplemental Material Table S1 - Primers used for QPCR analysis of gene expression

Human primers

	Forward	Reverse
**Adiponectin**	TCCTCACTTCCATTCTGACTG	GGACCAATAAGACCTGGATCT
**ALP**	TGCTCCCACGCGCTTGTGCCTGGA	CTGGCACTAAGGAGTTAGTAAG
**b-actin**	AGATCAAGATCATTGCTCCTC	ACTAAGTCATAGTCCGCCTA
**Fabp4**	AAAGTCAAGAGCACCATAACC	TTCAATGCGAACTTCAGTCC
**Leptin**	GGCTTTGGCCCTATCTTTTC	GGATAAGGTCAGGATGGGGT
**LPL**	AGGAGCATTACCCAGTGTCC	GGCTGTATCCCAAGAGATGGA
**OPN**	CACTCCAGTTGTCCCCACAGTAGA	GGCCTTGTATGCACCATTCAACTC
**OST**	AGGGCAGCGAGGTAGTGAAGA	AAGGGCAAGGGGAAGAGGAAAGAA
**PPARg2**	GCGATTCCTTCACTGATAC	TCAAAGGAGTGGGAGTGGTC
**Pref-1**	CTGCCTTCACGGACTCTG	GCCCGAACATCTCTATCACA
**Runx2**	GACAGCCCCAACTTCCTGT	CCGGAGCTCAGCAGAATAAT

Mouse primers

	Forward	Reverse
**Adiponectin**	AACTTGTGCAGGTTGGATGG	GCCCTTCAGCTCCTGTCATT
**ALP**	GGGACTGGTACTCGGATAACGA	CTGATATGCGATGTCCTTGCA
**b-actin**	GACGGCCAGGTCATCACTAT	CGGATGTCAACGTCACACTT
**Fabp4**	AGCCCAACATGATCATCAGC	TTTCCATCCCACTTCTGCAC
**Leptin**	GCCAGGCTGCCAGAATTG	CTGCCCCCCAGTTTGATG
**LPL**	ACAACCAGGCCTTCGAGATT	TCAGGCCAGCTGAAGTAGGA
**OPN**	GTATTGCTTTTGCCTGTTTGG	TGAGCTGCCAGAATCAGTCACT
**OST**	TGCTTGTGACGAGCTATCAG	GAGGACAGGGAGGATCAAGT
**PPARg2**	TGGGTGAAACTCTGGGAGATTC	AATTTCTTGTGAAGTGCTCATAGGC
**Pref-1**	CCTGGCTGTGTCAATGGAGT	CTTGTGCTGGCAGTCCTTTC
**Runx2**	TTTAGGGCGCATTCCTCATC	TGTCCTTGTGGATTAAAAGGACTTG

A.

Undifferentiated MSCs gene expression profile	Positive cellular markers and genes	Negative cellular markers and genes
MSCs	CD90, CD15, Sca1	
Hematopoietic lineage	CD34, CD68	CD19, CD31, CD79a, c-kit
Adipogenic lineage	Pref-1, FABP4, PPARγ(+/-)	LEP, ADIPOQ
Osteogenic lineage	OPN, ALP(+/-)	OST
Chondrogenic lineage	COL II, COL X (+/-)	ACAN

B.

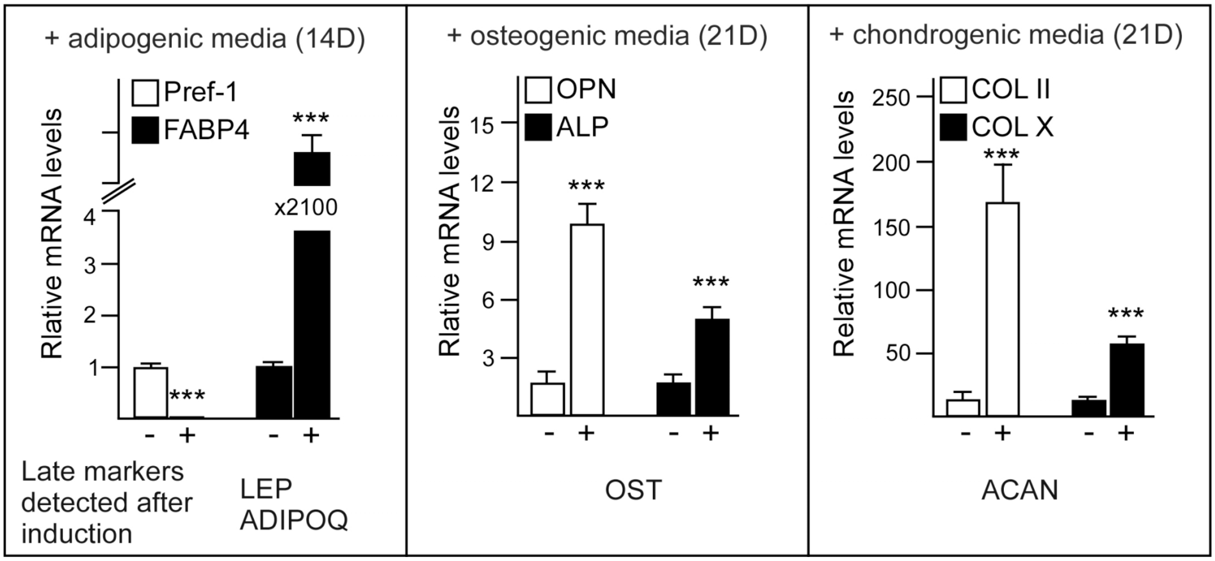

<u>Supplemental Material Figure S1 - Characterization of bone marrow-derived mouse MSCs.</u>

A) Gene expression profile of MSCs for multi-lineage progenitors and surface markers. The presence or absence of surface markers or transcripts specific to adipogenic, osteogenic and chondrogenic lineages was detected by real-time RT-PCR in undifferentiated MSCs, with the expression of each target gene normalized to β-Actin. A gene was considered not expressed in the cell preparation if its mean C_t value exceeded 38. The symbol (+/-) was assigned to a gene whose expression was low (mean C_t value exceeding 32), but was consistently detectable.

B) Multi-lineage assays. MSCs were differentiated into adipose, bone and cartilage throughout the experiments (up to Passage 6) to verify their multipotency. The expression level of specific lineage markers [adipogenic (Pref-1 and FABP4), osteogenic (OPN and ALP) and chondrogenic

(COL II and COL X)] was assayed by real-time PCR as previously described (Kirchner et al. 2010). Markers that were not significantly expressed in undifferentiated cells, were detectable in induced MSCs [adipogenic (LEP and ADIPOQ), osteogenic (OST) and chondrogenic (ACAN)]. Data were expressed as average fold change in expression in N=9 replicates ± SEM in differentiated relative to undifferentiated MSCs. Asterisks represent significant differences (*, $p<0.05$; **, $p<0.01$; ***, $p<0.001$). Supplemental Material Figure S1A represents the gene expression profiles of mouse bone marrow-derived MSC, hMSCs are not shown). These MSCs retained their capacity to differentiate into various lineages such as adipose, bone and cartilage regularly throughout the experiments (Supplemental Material Figure S1B). The multipotency of this MSC population was also assessed by analyzing mRNA levels for a panel of molecular markers (Kirchner et al. 2010). As expected, early adipocyte differentiation (2 days) was accompanied by induction of the PPARγ target gene, fatty acid binding protein 4 (FABP4, also known as aP2) and a decrease in mRNA levels of the adipocyte differentiation inhibitor, preadipocyte factor-1 (Pref-1, also called Dlk1, Delta-like protein 1) (Kirchner et al. 2010). Late events in adipogenesis (14 days) included the synthesis of adipocyte-secreted products such as leptin (LEP) and adiponectin (ADIPOQ) (Supplemental Material Figure S1B) (Kirchner et al. 2010). Osteogenic differentiation was measured by an early increase in bone specific alkaline phosphatase (ALP) and osteopontin (OPN) gene expression, followed by an increase in osteocalcin (OST) gene expression (Kirchner et al. 2010). Differentiation along the chondrogenic lineage was revealed by early increases in collagens II and X (COL II and COL X) and late aggrecan (ACAN) expression (Kirchner et al. 2010).

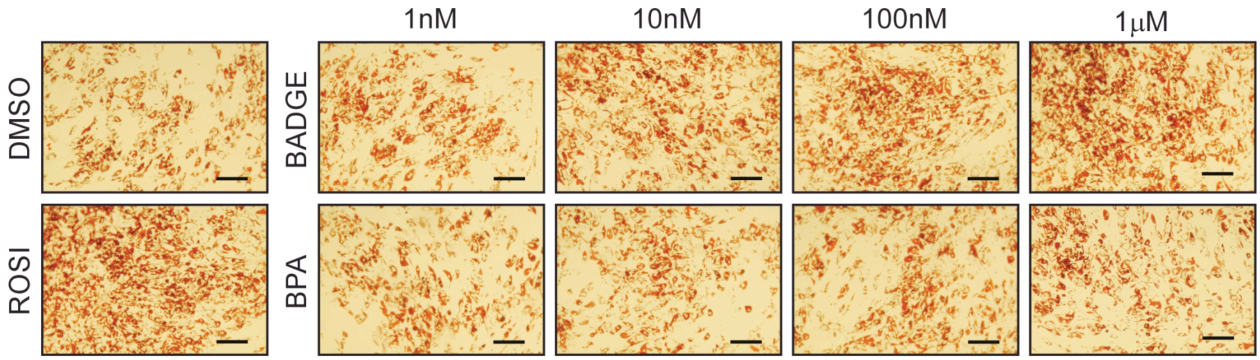

Supplemental Material Figure S2 - Dose response analysis of hMSCs adipogenic capacities to in vitro exposure to BPA and BADGE

Adipogenesis was induced in human MSCs in 6-well plates by the addition of the adipogenic cocktail (MDII) in the absence ((DMSO) or presence of ROSI (500 nM) or increasing dosages of BADGE and BPA (1 nM through 1 μM). After 14 days in culture, the cells were fixed and stained with Oil Red O. Ten random photographs were taken per well and converted to a black and white binary image using Image J (v1.36b Wayne Rasband) essentially as described (Kirchner et al. 2010). Lipid accumulation was measured by analyzing the percentage of the surface area in each photograph covered by stained cells in that particular image. The percentages obtained in the figures were averaged for each well independently. Data represent mean ± SEM from 3 independent experiments in duplicate. Scale bar, 200 μm.

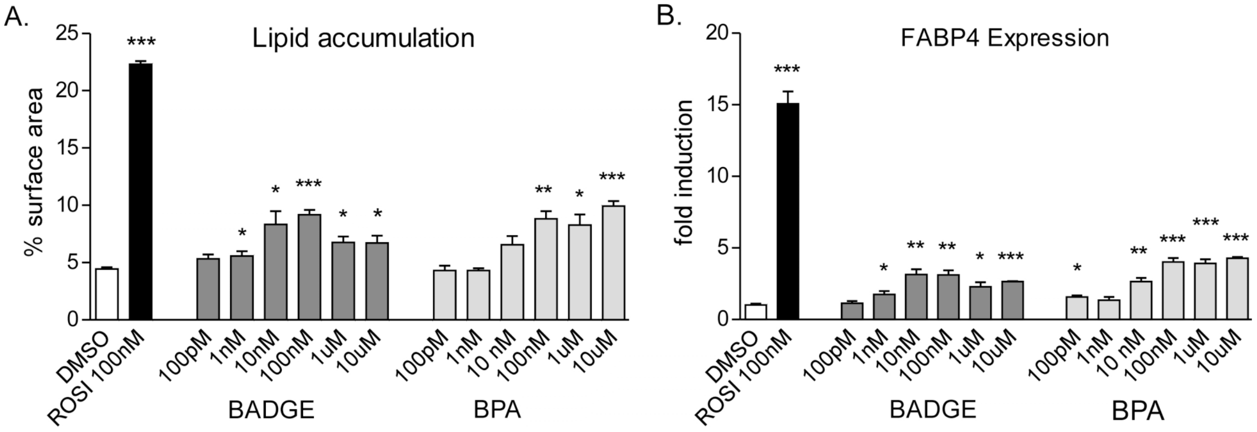

<u>Supplemental Material Figure S3 – Adipogenic response of 3T3-L1 preadipocytes to BADGE and BPA</u>

A) Adipogenesis assays. 3T3-L1 preadipocytes were treated with vehicle (DMSO), or the indicated concentrations of ROSI (positive control), BADGE and BPA. Adipogenic differentiation was revealed by staining with Oil Red O and lipid accumulation was quantified using Image J (Li et al. 2011). Data are shown as means ± S.E.M. for triplicate samples. 6 individual pictures were quantified and averaged for each sample. Data are expressed as average area fraction in n = 3 replicates ± SEM (n = 6 per well).

B) Gene expression. RNA was collected from the cells on day 7, followed by real time RT-PCR for gene expression analysis for FABP4. Data were presented as mean fold induction ± SEM compared with DMSO vehicle for triplicate samples. *P < 0.5, **P < 0.01 and ***P < 0.001 vs. vehicle controls.

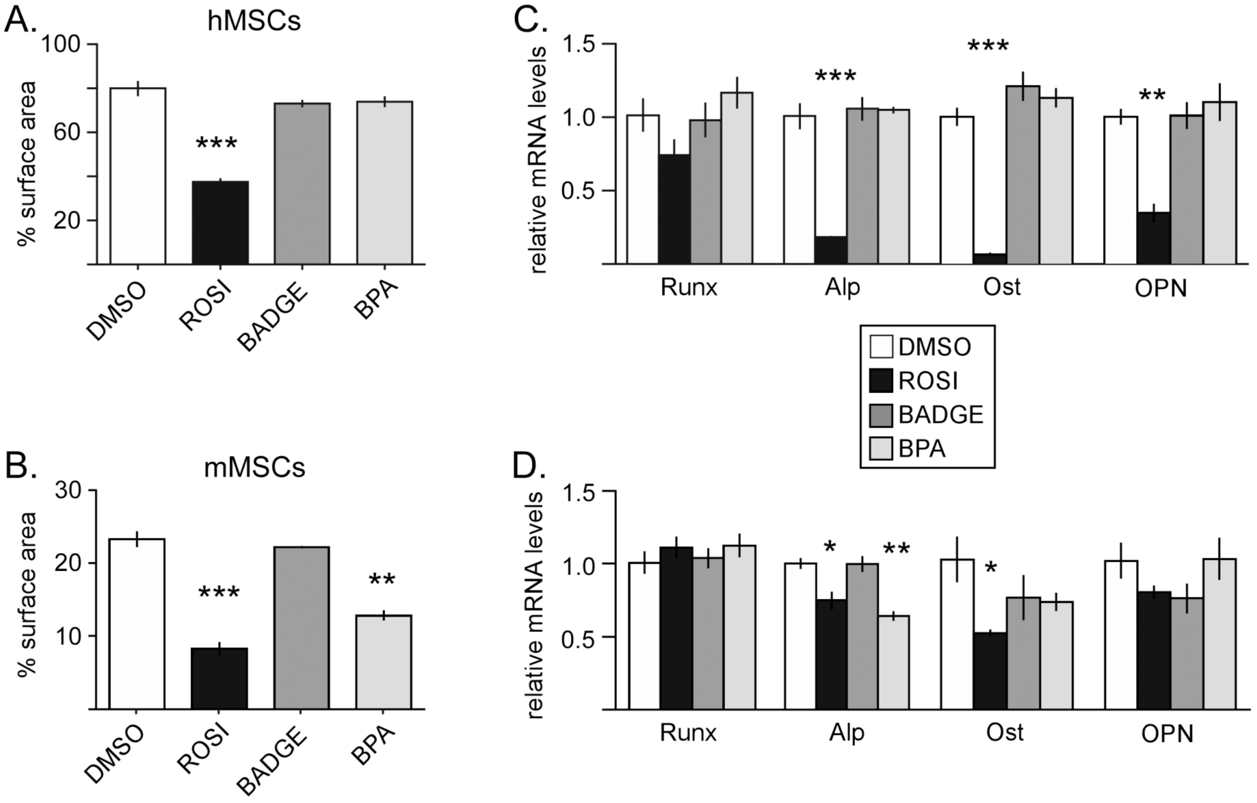

__Supplemental Material Figure S4 - In vitro effect of BADGE exposure on mMSC and hMSCs osteogenic capacities.__

A, B) Osteogenesis assays. Calcium accumulation in **A)** hMSCs or **B)** mMSCs. Osteogenesis was induced in MSCs by the addition of an osteogenic cocktail for 21 days in the absence (DMSO) or presence of ROSI (500 nM) (PPARγ), BADGE (100 nM) or BPA (100 nM). (N=3 wells per treatment). Undifferentiated cells were kept in basic MSCs expansion media, able to prevent differentiation, as a negative control (untreated cells). Calcium accumulation was stained by Alizarin Red-S and quantified with the Image J software.

C, D) Gene expression analysis. The expression of standard osteogenic markers was analyzed in **C)** hMSCs and **D)** mMSCs using QPCR of early markers (Runx2 and ALP) and late (OST and OPN) osteogenesis markers. Expression was normalized to β-actin. All data were expressed as average fold change in N=9 replicates ± SEM (N=3 wells per treatment in triplicates) relative to vehicle (DMSO) controls. Asterisks show significant differences (*, $p<0.05$; **, $p<0.01$; ***, $p<0.001$) vs. vehicle controls.

Literature Cited

Kirchner S, Kieu T, Chow C, Casey S, Blumberg B. 2010. Prenatal exposure to the environmental obesogen tributyltin predisposes multipotent stem cells to become adipocytes. Mol Endocrinol 24(3): 526-539.

Li X, Ycaza J, Blumberg B. 2011. The environmental obesogen tributyltin chloride acts via peroxisome proliferator activated receptor gamma to induce adipogenesis in murine 3T3-L1 preadipocytes. J Steroid Biochem Mol Biol 127(1-2): 9-15.
